# Ecological Conditions and Molecular Determinants Involved in *Agrobacterium* Lifestyle in Tumors

**DOI:** 10.3389/fpls.2019.00978

**Published:** 2019-07-30

**Authors:** Thibault Meyer, Clémence Thiour-Mauprivez, Florence Wisniewski-Dyé, Isabelle Kerzaon, Gilles Comte, Ludovic Vial, Céline Lavire

**Affiliations:** ^1^UMR Ecologie Microbienne, CNRS, INRA, VetAgro Sup, UCBL, Université de Lyon, Lyon, France; ^2^Biocapteurs-Analyses-Environment, Universite de Perpignan Via Domitia, Perpignan, France; ^3^Laboratoire de Biodiversite et Biotechnologies Microbiennes, USR 3579 Sorbonne Universites (UPMC) Paris 6 et CNRS Observatoire Oceanologique, Paris, France

**Keywords:** *Agrobacterium tumefaciens*, tumor lifestyle, crown gall, molecular traits, competition, plant defense

## Abstract

The study of pathogenic agents in their natural niches allows for a better understanding of disease persistence and dissemination. Bacteria belonging to the *Agrobacterium* genus are soil-borne and can colonize the rhizosphere. These bacteria are also well known as phytopathogens as they can cause tumors (crown gall disease) by transferring a DNA region (T-DNA) into a wide range of plants. Most reviews on *Agrobacterium* are focused on virulence determinants, T-DNA integration, bacterial and plant factors influencing the efficiency of genetic transformation. Recent research papers have focused on the plant tumor environment on the one hand, and genetic traits potentially involved in bacterium-plant interactions on the other hand. The present review gathers current knowledge about the special conditions encountered in the tumor environment along with the *Agrobacterium* genetic determinants putatively involved in bacterial persistence inside a tumor. By integrating recent metabolomic and transcriptomic studies, we describe how tumors develop and how *Agrobacterium* can maintain itself in this nutrient-rich but stressful and competitive environment.

## Introduction

The persistence of bacterial phytopathogens results from many factors including survival in natural habitats such as the soil, the rhizosphere, or the phyllosphere (van der Wolf and Boer, 2015), acclimation capacities to different lifestyles, and the ability to efficiently shift from one lifestyle to another (Sokurenko et al., 2006; Engering et al., 2013; Duprey et al., 2014; Wei et al., 2015). Survival in the rhizosphere implies abilities to resist both abiotic and biotic stress, and a high capacity to rapidly access nutritional sources (Ji and Wilson, 2002; Romanuk et al., 2009; Wei et al., 2015; Leonard et al., 2017). The pathogenic lifestyle generally involves bacterial growth inside the host and tight interactions with it. The success of the pathogen thus depends on its ability to face the conditions encountered inside the host, including plant defense mechanisms, availability of nutrient resources, and interactions with the host microbiota (Brader et al., 2017; Spanu and Panstruga, 2017; van der Does and Rep, 2017).

Members of the *Agrobacterium* genus are soil-borne bacteria able to live in the plant rhizosphere; they can be pathogenic when they harbor the Ti (Tumor-inducing) plasmid. This plasmid contains a DNA region (T-DNA) that can be transferred to plant cells and integrated into the plant genome. Briefly, the T-DNA genes are expressed by the infected plant, leading to hormone production which induces uncontrolled proliferation of plant cells (Drummond, 1979; Nester, 2014). Then the plant develops a tumor and is considered as suffering from crown gall disease. The T-DNA region also contains genes encoding opine biosynthesis. Opines are conjugates of amino acids and sugars or organic acids, and are specifically used as nutrients by agrobacteria harboring the Ti plasmid. Around 40 different types of opines have been characterized; some of them trigger the transfer of the Ti plasmid from one bacterium to another, enhancing pathogenicity and contributing to the persistence of pathogenic bacteria in the environment (Dessaux et al., 1998; Flores-Mireles et al., 2012). These properties confer opines a central role in the *Agrobacterium*-infected plant interaction (Guyon et al., 1980; Dessaux and Faure, 2018). According to the “opine concept,” the presence of opines improves the competitiveness of the bacteria able to catabolize them at the expense of the bacteria unable to do so. However, the ability to catabolize opines may not be sufficient to be competitive under detrimental environmental conditions. Tumor exploitation also implies dealing with plant defense mechanisms. Plant defense relies on signaling by chemical compounds such as salicylic acid (SA), jasmonic acid (JA), or ethylene that allow for the induction of pathogenesis-related proteins, some of which exhibit antimicrobial activities (Costet et al., 1999; Durrant and Dong, 2004). Even though plants initiate defense mechanisms against *Agrobacterium* (Deeken et al., 2006; Pitzschke, 2013; Shih et al., 2018), the bacterium is known to bypass those defenses to durably settle in tumors (Lee et al., 2009; Someya et al., 2013; Nonaka and Ezura, 2014; Nonaka et al., 2017). It should be emphasized that *Agrobacterium* is one of the only bacterial phytopathogens that exploits its host for niche construction instead of killing it (Leonard et al., 2017).

Non-pathogenic *Agrobacterium* strains have also been isolated from tumors (Bélanger et al., 1995; Llop et al., 2009; Shams et al., 2012). Some strains could be mutants of the initial tumor-inducing strain (e.g., following spontaneous deletions inside pTi), but most of these non-pathogenic isolates may have an environmental origin. Tumors have also been reported to host other microorganisms (Faist et al., 2016). Hence, even in an opine-rich tumor environment and beyond its ability to use opine as a nutrient, *Agrobacterium* needs additional determinants to compete with other microorganisms and durably settle.

The induction of the pathogenicity program, as well as the process of genetic transformation leading to tumor formation, has been extensively studied (Lacroix and Citovsky, 2013; Nester, 2014; Gelvin, 2017). The natural ability of *Agrobacterium* to genetically transform plants and its use as a tool for genetic engineering are also well documented (Hwang et al., 2015; Krenek et al., 2015). Moreover, the transition between the rhizospheric and tumor lifestyles, which represents a critical step in the establishment of crown gall disease, has recently been reviewed (Barton et al., 2018). As *Agrobacterium* pathogenicity and perennial soil contamination are directly linked to its success in maintaining a long-term association with its host plant, we chose to focus this review on the bacterial ability to durably exploit the specific tumor environment. We therefore summarized knowledge about *Agrobacterium* settlement and the subsequent changes triggered in the host plant, and focused on the molecular determinants that allow *Agrobacterium* to survive in tumors, exploit this environment, and compete with other microorganisms encountered in tumors.

## Tumor Development Causes Stress in Bacterial and Plant Cells

Once the T-DNA is integrated, tumor development is initiated and the physico-chemical parameters of the tumor evolve with time and with the host plant biotic and abiotic environment (Hwang et al., 2015). We focused on the specific conditions encountered in tumors and on the bacterial genes that are likely relevant for thriving in that special environment and modifying it.

### Role of Hormones

The role of jasmonic acid (JA) in tumor development is not formally established and seems to differ according to the plant species. No increase in JA production was observed during tumor development in *Arabidopsis thaliana* (Lee et al., 2009), whereas transient JA accumulation occurred in 1-week-old *Ricinus communis* tumors (Veselov et al., 2003). By contrast, the role of auxin, cytokinin, and ethylene in tumor proliferation and development has been established (for a review see Gohlke and Deeken, 2014). Native plant cells contain genes responsible for the synthesis of these types of hormones, but transformed cells contain additional T-DNA-encoded genes for auxin and cytokinin biosynthesis, i.e., *iaaH* and *iaaM* for auxin, and *ipt* for cytokinin (Skoog and Miller, 1957; Zhu et al., 2000). Tumors contain higher levels of auxin and cytokinin than non-infected plant stems; concentrations vary according to the plant and the age of the tumor (Veselov et al., 2003; Lee et al., 2009; Gohlke and Deeken, 2014). *Agrobacterium* cells can also produce hormones. Indeed, two genes have been proposed to be involved in cytokinin production of *Agrobacterium*: *miaA* (*atu2039*) located on the C58 circular chromosome (Gray et al., 1996) and *tzs* (*atu6164*) located on several pTi including the nopaline C58-pTi (Hwang et al., 2010). Both genes are expressed in C58-induced tumors (González-Mula et al., 2018), suggesting that bacteria produce cytokinin in that environment. *Agrobacterium* can synthesize another hormone, the indole-3-acetic acid (IAA of the auxin family). Although the genetic determinants of IAA synthesis remain unknown, this property is not T-DNA encoded, as the non-oncogenic strain GV3101 can also produce IAA (Lee et al., 2009).

**Figure 1:**
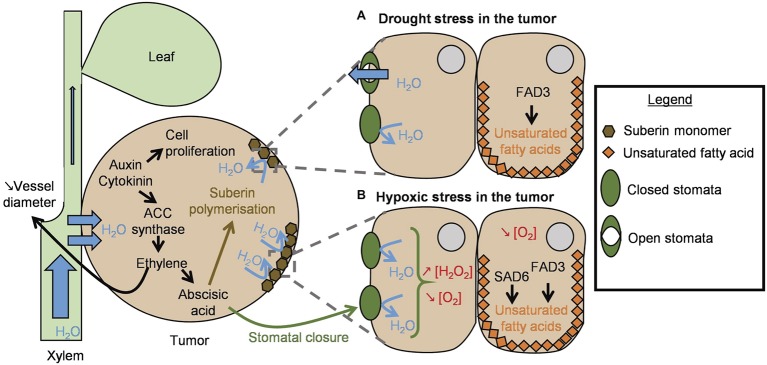
Schematic representation of the plant reactions to the stresses caused by tumor development. T-DNA integration into the plant genome induces auxin and cytokinin production. High concentrations of these two phytohormones accelerate cell proliferation and tumor growth. ACC synthase expression is also induced and triggers ethylene production. Ethylene has two main roles in the tumor: it reduces plant vessel diameter around the tumor to ensure its hydration, and it triggers abscisic acid synthesis. The latter induces the polymerization of suberin that forms a protective layer around the tumor. **(A)** Drought stress in the tumor: under drought stress, FAD3 produces α-linolenic acid, an unsaturated fatty acid, to maintain lipid membrane integrity. **(B)** Hypoxic stress in the tumor: when the drought stress is more severe (i.e., in old tumors), abscisic acid triggers stomatal closure. This implies H_2_O_2_ production and leads to a decreased oxygen rate. Under low oxygen, SAD6 also contributes to the production of unsaturated fatty acids to maintain lipid membrane integrity in the transformed cells.

High cytokinin and auxin levels enhance the activity of 1-aminocyclopropane-1-carboxylate (ACC) synthase, the key enzyme of ethylene synthesis in plants (Adams and Yang, 1979). Ethylene has been proposed to be involved in the high amount of vascularized tissues in tumors at the detriment of aerial part, leading to the water-flow priority to tumor cells over the host shoot (i.e., “Gall-constriction hypothesis”; Aloni, 1995; Aloni et al., 1998). Indeed, the comparison of tumors induced by *A. tumefaciens* strain C58 in wild-type tomato and in *never*-*ripe* mutants (sensitive and insensitive to ethylene, respectively) revealed that the mutant plants had smaller tumors and more vascularized tissues than the wild-type plants (Aloni et al., 1998). Thus, ethylene plays a central role in tumor water supply (Figure 1A). *Agrobacterium* growth is not affected by ethylene (strain C58; Nonaka and Ezura, 2014) and *Agrobacterium* cannot directly affect the ethylene content. While some plant-associated bacteria (either phytobeneficial or phytopathogenic) can degrade the immediate precursor of ethylene, ACC, *Agrobacterium* fails to do so, because it lacks the gene encoding the ACC-deaminase enzyme (Someya et al., 2013; Bruto et al., 2014; Nonaka and Ezura, 2014).

SA take part in plant defense mechanisms called systemic acquired resistance (SAR) and local acquired resistance (LAR) by triggering the synthesis of pathogenesis-related proteins (Costet et al., 1999; Durrant and Dong, 2004). However, transcriptomic analyses have demonstrated that genes involved in plant defense were under-expressed in the early stages of infection by *Agrobacterium* (Veena et al., 2003; Lee et al., 2009). In 5-week-old *A. thaliana* tumors, the activation of the SA-dependent defense signaling pathway was not triggered despite an increase in SA levels. This observation was attributed to the high level of auxin in these tumors (Lee et al., 2009). SA can inhibit bacterial growth when added to the culture medium (Yuan et al., 2007; Anand et al., 2008). The SA inhibitory concentration could vary according to bacterial growth conditions: 5 μM in a minimal culture medium (Yuan et al., 2007) as compared to 200 μM in an enriched one (Anand et al., 2008). As for IAA, its effect is dose-dependent: it promotes bacterial growth at low concentrations (0.8 μM) but shows deleterious effects at high concentrations (200 μM) (Liu and Nester, 2006; Gohlke and Deeken, 2014). Hence, *Agrobacterium* could first benefit from the presence of IAA, but could be locally sensitive to high levels of this compound likely to impede its development as the tumor grows (Gohlke and Deeken, 2014). Thus, in the tumor tissue, ethylene circumvents its classical role in defense response and highly participates in tumor development together with cytokinin and auxin.

### Drought

The water advantage conferred by ethylene through its influence on xylem vessel diameter is further counterbalanced by an increased water loss: the massive proliferation of tumor cells causes epidermal rupture and uncontrolled evaporation of the water contained in the tumor (Figure 1; Schurr et al., 1996). In this case, ethylene also plays a crucial role in tumor drought tolerance, as proposed earlier (Efetova et al., 2007) and as summarized in Figure 1A. Cytokinins and auxins trigger ethylene synthesis that in turn induces abscisic acid (ABA) synthesis in the tumor. ABA induces suberin polymerization to create a protective layer around the tumor and avoid its desiccation (Veselov et al., 2003; Efetova et al., 2007; Gohlke and Deeken, 2014). In response to the drought stress mediated by ABA signaling, enhancement of fatty acid desaturase activity is also observed in tumor cells, to synthesize α-linolenic acid and ensure lipid membrane integrity (Klinkenberg et al., 2014). Fatty acid desaturase 3 (FAD3) plays an important role in tumors: infection of *A. thaliana* FAD3 mutants by *Agrobacterium* strain C58 at low humidity rates revealed that *fad3-2* mutant tumors were smaller than wild-type ones. This demonstrates that FAD3-synthesized α-linolenic acid can maintain the lipid membrane integrity of plant cells under drought conditions (Figure 1; Klinkenberg et al., 2014).

In order to maintain homeostasis, bacteria use two main strategies, i.e., accumulation of osmoprotectants and production of exopolysaccharides (EPSs) (Weiner et al., 1995; Dandapath et al., 2017). Osmoprotectants are compounds that accumulate into the cell to protect it from different stresses such as drought, dehydration, or the presence of oxygen radicals. *Agrobacterium* can take up or synthesize a large range of compounds previously described as being involved in osmoprotection (Kempf and Bremer, 1998; Panikulangara et al., 2004; Bougouffa et al., 2014). These compounds include glycine betaine and choline (Boncompagni et al., 1999), gamma-butyrobetaine (Nobile and Deshusses, 1986), proline (Haudecoeur et al., 2009b), sucrose and trehalose (Ampomah et al., 2013), palatinose (De Costa et al., 2003), melibiose, raffinose, and stachyose (Meyer et al., 2018b). Bacterial genes allowing for melibiose, raffinose, and stachyose uptake are expressed in mature C58-induced tumors (González-Mula et al., 2018) where they could allow for bacterial osmoprotection. However, the role of these compounds in bacterial osmoprotection has not been assessed yet. A direct role of gamma-butyrobetaine, glycine betaine, and choline in *Agrobacterium* osmoprotection has been described only *in vitro* (notably for C58), but so far no study has investigated their role *in planta* (Nobile and Deshusses, 1986; Boncompagni et al., 1999).

Biosynthesis of EPS is the second way of resisting high or low environmental osmolarities. *Agrobacterium* can synthesize diverse kinds of EPS: β-1-2-glucan (*atu2728–atu2730*) (Cangelosi et al., 1990), curdlan (*atu3355–atu3357*) (Stasinopoulos et al., 1999; McIntosh et al., 2005; Lassalle et al., 2011; Ruffing and Chen, 2012), succinoglycan (*atu4049–atu4060; atu4166; atu3325–atu3327*) (Tomlinson et al., 2010), unipolar polysaccharide (*atu1235–atu1240; atu0102*), cellulose (*atu3303–atu3309*) (Matthysse, 2018). Among the EPS, only curdlan is specific to the “*A. fabrum*” species (Lassalle et al., 2011, 2017). A role in osmoprotection has only been described for β-1-2-glucan, which is produced in response to low-osmolarity conditions in different microorganisms including *Agrobacterium* (Cangelosi et al., 1990; Ingram-Smith and Miller, 1998; Domínguez-Ferreras et al., 2006). Some *Agrobacterium* genes involved in EPS (curdlan) production are expressed in C58-induced tumors (González-Mula et al., 2018) where they may serve as osmoprotectants. Alternatively, they could be useful for cell adhesion, as some of these EPSs are involved in that process (for a review see Matthysse, 2018). The precise role of EPS inside tumors thus remains to be deciphered.

### Oxidative and Hypoxic Stress

One of the first plant responses to pathogen perception is the production of reactive oxygen species (ROS), like superoxide (O_2_^−^), or hydrogen peroxide (H_2_O_2_) (Torres et al., 2006; Lee et al., 2009). *Agrobacterium* strain C58 can detoxify these toxic compounds thanks to proteins encoded by chromosomal genes. Firstly, it harbors three genes encoding superoxide dismutase: *sodBI* (*atu0876*), *sodBII* (*atu4583*), and *sodBIII* (*atu4726*) (Saenkham et al., 2007). The functional analysis of these three enzymes revealed that SodBI is mainly responsible for transforming O_2_^−^ into O_2_ and H_2_O_2_. The *sodBI* mutant and the triple superoxide dismutase mutant were less proficient than the wild-type in inducing *vir* genes and consequently displayed a reduced ability to induce tumor formation (Saenkham et al., 2007). Secondly, *Agrobacterium* can degrade H_2_O_2_ to H_2_O and O_2_ thanks to the KatA catalase (*atu4642* carried by strain C58 linear chromid – i.e., linear chromosome) (Xu and Pan, 2000). A C58-derivative *katA* mutant was highly affected in its capacity to form tumors. It has been suggested that *Agrobacterium* catalase expression prevents the infected plant from activating the hypersensitive response (HR), allowing for the bacterium to settle and to form tumors without risking plant tissue necrosis (Xu and Pan, 2000; Gohlke and Deeken, 2014). *katA* expression is induced by OxyR upon H_2_O_2_ perception (Nakjarung et al., 2003). Conversely, in *Agrobacterium* strain C58, the global regulator LsrB was recently shown to negatively regulate *katA* transcription (Tang et al., 2018). The ability to cope with oxidative stress likely participates in the long-term efficient interaction. Five-week-old *R. communis* tumors were reported to contain hypertrophied non-functional stomata at the tumor surface that increased tumor water loss (Figure 1B; Schurr et al., 1996). The ABA drought stress pathway is involved in stomatal closure through its influence on H_2_O_2_ production (Zhang et al., 2001). Accordingly, although H_2_O_2_ accumulation was impeded by the bacteria at the beginning of the infection process (3 h and 6 days after inoculation), it accumulated later during tumor development (5 weeks after inoculation) (Lee et al., 2009). To determine if the differences in H_2_O_2_ levels are due to lower bacterial degradation and/or greater production by plants or by bacterial cells, it would be relevant to study the expression of the bacterial genes *sodB*, *katA,* and their regulator, and also H_2_O_2_ accumulation during tumor development.

The oxygen rate inside tumors decreases, and hypoxic stress has consequences on the plant metabolism, notably on the fatty acid metabolism. In these conditions, stearoyl ACP desaturase 6 (SAD6), which is synthesized only in a hypoxic environment, maintains plant cell membrane integrity by catalyzing the first step of fatty acid biosynthesis (Figure 1B; Klinkenberg et al., 2014; Kerpen et al., 2019). In accordance, in 5-week-old *A. thaliana* tumors, genes involved in photosynthesis were under-expressed, whereas genes involved in glycolysis and fermentation were over-expressed (Deeken et al., 2006). The authors suggested that transformed plant cells switch from an autotrophic metabolism to a heterotrophic one and that oxygen availability is limited, at least in some parts of the tumor. The low oxygen content of tumors has been recently confirmed in 14- and 28 day-old *A. thaliana* tumors induced by *Agrobacterium* strain B6 (Kerpen et al., 2019). Under low oxygen conditions, some *Agrobacterium* species are facultative denitrifiers (Baek and Shapleigh, 2005). A nitrate reductase (NapAB: Atu4408–Atu4409) allows them to reduce nitrate into nitrite (NO_2_^−^), which is further converted into nitric oxide (NO) by a nitrite reductase (NirK: Atu4382). Finally, an NO reductase (NorB: Atu4388) catalyzes the transformation of NO into nitrous oxide (N_2_O), which accumulates in the absence of an N_2_O reductase (encoded by the *nosZ* gene) (Kampschreur et al., 2012). The *norB* gene was expressed in a context of agroinfiltration in *A. thaliana* leaves, and a transcriptomic study comparing tumors and culture media suggests that partial denitrification occurs in tumors (Baek and Shapleigh, 2005; González-Mula et al., 2018). These results indicate that on the one hand, denitrification can participate in *Agrobacterium* survival in tumors by providing energy in the absence of oxygen, and on the other hand, NO degradation by *Agrobacterium* NO reductase can also participate in the bypassing of plant defenses mediated by NO (Delledonne et al., 1998; Leitner et al., 2009; Bellin et al., 2013).

## Bacteria Find Trophic Resources and Toxic Compounds Inside Tumors

### Tumor Metabolite Content

Nutrient availability is crucial for bacterial development inside tumors. A tumor is a highly vascularized metabolite sink supplied by water and minerals (mainly conducted *via* the xylem) and by sugars and other biosynthesized carbonated compounds (transported *via* the phloem) (Gohlke and Deeken, 2014). Apart from hormones, ABA and opines, whose presence in the tumor has been specifically analyzed at different times after inoculation, only a few studies have dealt with metabolite analyses (Deeken et al., 2006; Simoh et al., 2009; González-Mula et al., 2019). Moreover, these studies used different bacterial strains, plant models (*A. thaliana*, *Brassica napa*, *Solanum lycopersicum*), and analytical methods (targeted or global analyses based on NMR or GC-TOF-MS). Overall, as in non-infected tissues, the main primary metabolites in tumors were sugars, organic acids, and amino acids (Deeken et al., 2006; González-Mula et al., 2019). In addition to sucrose and glucose, and as compared to non-infected stems, 5-week-old *A. thaliana* tumors contained a high concentration of amino acids (glutamine, serine, asparagine, glutamic acid, threonine, proline, aspartic acid, alanine, valine, isoleucine, leucine, histidine, and arginine) (Deeken et al., 2006). Among sugars, organic acids and amino acids, and in contrast to *A. thaliana* tumors, only pyruvate and proline significantly accumulated in *S. lycopersicum* tumors (González-Mula et al., 2019). *γ*-hydroxybutyric acid (GHB) also slightly accumulated in *S. lycopersicum* tumors (González-Mula et al., 2019). Among nitrogen compounds, GABA and α-aminoadipinic acid were more abundant in infected tissues (Deeken et al., 2006; González-Mula et al., 2019). Secondary metabolites, some of which can limit bacterial growth (Del Valle et al., 2016; Wang et al., 2018) can also be detected in tumors: flavonoids such as quercetin and kaempferol, phenylpropanoids and its derivatives (ferulate, sinapoyl- and coumaroyl-malate, 3-caffeoylquinate), and glucosinolate or its derivatives are also accumulated in *B. napa* or *S. lycopersicum* tumors (Simoh et al., 2009; González-Mula et al., 2019).

Metabolites are not uniformly distributed across tumors, and both their amount and their localization vary during tumor development. For example, the hexose level was found higher in the center of 3-week-old *R. communis* tumors than in their periphery. Conversely, sucrose and proline were more abundant in the periphery of the tumor than in its center in 6-week-old tumors (Wächter et al., 2003). This makes sense since sucrose is more easily transportable and assimilable under drought conditions (Singh and Maclachlan, 1983; Wobus and Weber, 1999; Deeken et al., 2006). Those gradients are proposed to be linked mainly to tumor expansion and also to turgor pressure and osmoprotection. The relative abundance of metabolites also depends on the opine-type of the *Agrobacterium* strain (Simoh et al., 2009): *B. napa* tumors induced by strains harboring a “nopaline-type” or a “octopine-type” Ti plasmid differed in flavonoids, phenylpropanoid derivatives, sugar, organic and amino acid contents. However, no difference was reported for the precursors of nopaline and octopine (i.e, arginine and respectively α-ketoglutarate and pyruvate; Simoh et al., 2009). Additional metabolomic studies of tumors would provide further insights into the *Agrobacterium*-plant interaction. Nevertheless, it appears that bacteria can use plenty of metabolites as nutrient resources in tumors and have to cope with toxic compounds.

### Opine Catabolism and Tumor Colonization

Pathogenic agrobacteria can catabolize opines using genes carried by their Ti plasmid. Opines are mainly used as carbon and nitrogen sources, but can also be used as phosphate or sulfur sources (Dessaux et al., 1998; Flores-Mireles et al., 2012; Dessaux and Faure, 2018). One of the features of the Ti plasmid is that it confers the bacterium that harbors it the possibility to catabolize a specific type of opine determined by the Ti plasmid-type (Gordon and Christie, 2014). The genes involved in the uptake of the well-described opines are actively studied (Marty et al., 2016, 2019; Dessaux and Faure, 2018). Among them, in *Agrobacterium* strain C58, *nocT* (*atu6027*) encodes a periplasmic binding protein (PBP) involved in the binding of nopaline and pyronopaline (Lang et al., 2014). The *ocd* (*atu6016*) gene encodes an ornithine cyclodeaminase involved in nopaline and pyronopaline degradation (Sans et al., 1987). *nocT* and *ocd* mutant strains are unable to use and assimilate these two opines. In competition assays for tumor niche occupancy, the wild-type strain outcompeted the two mutant strains, indicating that opines favor the survival of bacteria able to catabolize them under competitive conditions (Lang et al., 2014).

Interestingly, after individual inoculation, the wild-type and the two *nocT* and *ocd* mutants reached the same population level (Lang et al., 2014). This finding implies that the tumor provided other carbon sources that supported the growth of the two mutants. Accordingly, metabolomics studies and the *Agrobacterium* tumor transcriptome revealed a large diversity of available carbon sources together with the up-regulation of numerous and diverse genes involved in metabolism (Deeken et al., 2006; Simoh et al., 2009; González-Mula et al., 2019).

### Additional Nutrient Resources

Annotation of the *Agrobacterium* strain C58 genome predicted a large set of transporters putatively involved in the import of plant compounds, composed of 190 ABC (*ATP-binding cassette transporters*) and 3 TRAP (Tripartite ATP-independent periplasmic) transporters (Goodner et al., 2001; Wood et al., 2001). However, only a few of them has been characterized. The presence of multiple transporters for one substrate could be beneficial for the fitness of *Agrobacterium* in competition with other microorganisms and a redundancy of transporters importing plant substrates is found in *Agrobacterium*. For example, the *gguABC*-*chvE* (*atu2345–atu2348*) and the *gxySBA* (*atu3574–atu3576*) clusters are two distinct ABC transporters allowing for D-glucose, D-xylose, D-fucose, D-galactose, D-glucosamine, and L-arabinose uptake in *Agrobacterium* (Zhao and Binns, 2011, 2014). D-glucosamine, D-fucose, and D-glucose transport were shown experimentally dependent on both transporters (Zhao and Binns, 2014). The presence of additional transporters was suggested for L-arabinose, D-xylose, and D-galactose, as the strains deleted for genes encoding both transporters (*gguABC-chvE* and *gxySBA*) could still grow on these compounds (Zhao and Binns, 2014). In the case of D-galactose, the *mel* operon (encoding an ABC transporter) was suggested as the additional transport system in *Agrobacterium* (Meyer et al., 2018b). Expression of the *Agrobacterium mel* operon was induced inside the tumor (González-Mula et al., 2018) and a deletion mutant of the PBP uptake gene of this operon (responsible for melibiose, galactinol, raffinose, and stachyose uptake) was less competitive than the wild-type strain (Meyer et al., 2018b). However, it is not yet known whether the *mel*-imported compounds are used for osmoprotection and/or nutrition.

Sugars such as glucose, fructose, sucrose, xylose, fucose, and arabinose are commonly degraded by plant-associated bacteria (Lugtenberg et al., 1999; Gunina and Kuzyakov, 2015). Only a few catabolic pathways involved in plant compound assimilation have been described in *Agrobacterium*, mainly in strain C58. The *pycA* gene (*atu2726*) encodes a glucose-6P isomerase essential for *Agrobacterium* growth on sucrose, fructose, and glucose, which are abundant compounds in the tumor (González-Mula et al., 2019). Accordingly, a *pycA* deletion mutant was affected in its capacity to compete with a wild-type strain in the tumor. A similar result is obtained with a mutant strain unable to degrade GHB. This compound is degraded in *Agrobacterium* by BlcRABC (formerly known as the AttJKLM system, Atu5136-Atu5139; Carlier et al., 2004). The BlcRABC mutant was outcompeted by the wild-type strain in tumors (Haudecoeur et al., 2009a; González-Mula et al., 2019).

Some bacterial pathogens feed on plant cell wall degradation products (Reverchon and Nasser, 2013). *Agrobacterium* strain C58 was suggested to be able to degrade plant cell wall. For example, thanks to XynA (Atu2371), C58 strain is able to grow on xylan, a common component of plant cell wall (Mathews et al., 2019). The polygalacturonase PglA (Atu3129) and PglB (Atu4560) are involved in polygalacturonic acid degradation (Mathews et al., 2019). The *pglA* gene was suggested to form an operon with *picA*, a gene that is induced by plant extracts (Rong et al., 1991; Mathews et al., 2019). Interestingly, although galacturonic acid is detected in tumors (González-Mula et al., 2019), only *picA* is overexpressed (González-Mula et al., 2018). *Agrobacterium* can import galacturonic acid owing to the *gaaPQM* operon (*atu3135-atu3137*) which also allows for glucuronic acid uptake, yet to a lesser extent. Both organic acids can be used *in vitro* for growth (Zhao and Binns, 2016). Galacturonic acid is transformed into α-ketoglutarate by enzymes encoded by *atu3138-atu3143* (for details about galacturonic acid degradation, see Boer et al., 2010; Andberg et al., 2012; Taberman et al., 2014a,b; Zhao and Binns, 2016).

*Agrobacterium* strain C58 possesses other genetic determinants that allow it to grow on plant compounds but whose role and importance in tumor colonization have not yet been specifically studied. For example, *Agrobacterium* possesses a functional palatinose and trehalose uptake and degradation system (Ampomah et al., 2013). The *thuEFGKAB* operon (also known as the *palEFGKAB operon; atu3338-atu3343*) is composed of genes encoding an ABC transporter (*thuFGK*) linked to a gene encoding a PBP (*thuE*), and the *thuB* and *thuA* genes are those involved in the degradation process (Ampomah et al., 2013). Deletion of the genes coding for the ABC transporter abolished *Agrobacterium* growth on palatinose (De Costa et al., 2003), while maltitol, trehalose, and isomeric forms of sucrose were still imported and used for growth, again suggesting transporter redundancy. The *thuEFGK* deletion mutant is not required for tumor formation but as often, its role in the competitive colonization of the tumor has not been assessed yet. In addition to the above-described genes, other genes appear to be up-regulated in the tumor as compared to minimal media, and could be involved in the metabolism of carbon sources according to their annotation (González-Mula et al., 2018, 2019). Thus, *Agrobacterium* seems to be capable of using a large set of plant compounds to sustain its growth inside the tumor. Besides the functional characterization of genes involved in the uptake and catabolism of these plant compounds, additional efforts to decipher their roles not only *in vitro*, but also *in planta* and in competitive plant colonization are needed. Finally, whether *Agrobacterium* displays a preference for opines rather than for other carbon sources available in the tumor remains an open question.

### Toxic Compounds

The host plant also produces potential antibacterial compounds in the tumor, including phenolic compounds (Schwalm et al., 2003; Cushnie and Lamb, 2005; Deeken et al., 2006; Simoh et al., 2009; Shi et al., 2016; González-Mula et al., 2019). The VirH2 protein, an *O-*demethylase whose pTi-located gene is highly expressed in tumors, plays a role in the transformation of ferulic acid (highly present in tumors) into caffeic acid, a less toxic phenolic compound (Brencic et al., 2004; González-Mula et al., 2019). Some chromosomal genes are also involved in phenolic compound degradation. In “*A. fabrum*” species, the SpG8-1b genomic region is involved in ferulic acid, caffeic acid, and *p*-coumaric acid degradation (Lassalle et al., 2011; Campillo et al., 2014; Baude et al., 2016). In addition to detoxifying these phenolic compounds, some of these genetic determinants extend the metabolic versatility of “*A. fabrum*” by weakly sustaining growth (Campillo et al., 2014). Their expression is tightly regulated by the HcaR repressor, and this regulation is important for bacterial fitness in the tumor (Meyer et al., 2018a). Other non-characterized pathways putatively annotated as phenolic compound degradation pathways are present in other *Agrobacterium* species, highlighting the importance of this function (Lassalle et al., 2017).

The action of efflux pumps is another mechanism allowing *Agrobacterium* survival in the presence of toxic compounds. In *Agrobacterium* strain C58, three operons have been described, all located on the linear chromid. The AcrABR efflux system (*atu3003–atu3001*) was shown to export numerous toxic compounds and to confer resistance to high concentrations of these compounds (Nuonming et al., 2018). The EmrBAR efflux pump (*atu4479–atu4476*) has recently been shown to confer resistance to toxic compounds and to be induced by indole and flavonoids among which quercetin, a compound detected in tumor tissues (see section “Tumor Metabolite Content”; Simoh et al., 2009; Lee et al., 2015; Khemthong et al., 2019). Both of these systems could be useful for bacterial survival in tumors. However, the corresponding genes (*acrABR* and *emrBAR*) were not up-regulated in 3-week-old *A. thaliana* tumors as compared to the culture medium (González-Mula et al., 2018). This study rather revealed overexpression of *tetR/tetA* (*atu4205–atu4206*), which encodes an efflux pump conferring resistance to tetracycline (González-Mula et al., 2018). To date, the plant compounds genuinely exported by TetR/TetA remain unknown (Luo and Farrand, 1999; Lassalle et al., 2011).

## *Agrobacterium* Faces Microbial Competition in the Tumor

*Agrobacterium* long-term colonization of the tumor is partly dependent on competitions for opine nutritive resource, either between strains of *Agrobacterium* or with other microorganisms. Inside tumors, opines are public goods that are produced by the infected plant cells elicited by virulent *Agrobacterium*. However, opines can be shared within all the opines-catabolizing populations, not necessarily the virulent strains that originally induced tumors (For a review, see Platt et al., 2014). Tumors were shown to contain avirulent but opines-catabolizing *Agrobacterium* strains (either from environmental origin or derived from the strain inducing the tumor) (Bélanger et al., 1995; Llop et al., 2009). Those strains can be considered as cheaters (not expressing virulence genes but using opines), that may outcompete the *Agrobacterium* virulent population, burdened by the cost of infecting the plant due to the expression of virulence genes (Platt et al., 2012a,b). Virulence genes are still highly expressed in *A. thaliana* tumors, even 3 weeks post inoculation (González-Mula et al., 2018). The cost of the pTi plasmid could thus lead to plasmidless-genotypes dominance inside tumors. However, in the opine-rich tumor environment, the cost of the Ti plasmid is counterbalanced by opine benefits (as reviewed by Platt et al., 2014). In any case, competition for opine and tumor colonization occurs between *Agrobacterium* strains. Such a competition would have consequences on the persistence of the pTi rather than on *Agrobacterium* itself.

Besides, the presence of different *Agrobacterium* strains, it was recently reported that the microbial community of natural *Vitis vinifera* tumors caused by *Allorhizobium vitis* (another tumorigenic *Rhizobiaceae* species previously known as *Agrobacterium vitis*) contains more than 150 species, among which members of the *Pseudomonas*, *Sphingomonas*, *Erwinia*, and *Bradyrhizobium* genera (Faist et al., 2016). Unfortunately, no such global analyses have been conducted for *Agrobacterium*-induced tumors. Nonetheless, some studies report that tumors can harbor bacteria belonging to the *Corynebacterium* or the *Arthrobacter* genera or even to the *E. meliloti* species, a well-known plant symbiont (Tremblay et al., 1987; Nautiyal and Dion, 1990; Moore et al., 1997). The tumor environment can also shelter fungal species that are also opine degraders, such as *Cylindrocarpon destructans*, *C. heteronema*, and *Fusarium solani* (Beauchamp et al., 1990). Moreover, in the tumor, some *Pseudomonas putida* strains can catabolize mannopine, and some fungi catabolize mannopine and succinamopine (Nautiyal and Dion, 1990). *Pseudomonas* spp. can take the advantage over *Agrobacterium* when co-cultured in a medium supplemented with octopine, indicating that *Pseudomonas* spp. could use octopine more efficiently than *Agrobacterium in vitro* (Bell et al., 1990). In addition to metabolic abilities, other genetic determinants may allow *Agrobacterium* to compete with the tumor microbiota to durably settle in this environment.

The Type VI Secretion System (T6SS) described in several *Agrobacterium* strains could represent one such feature (Wu et al., 2012, 2019; Lin et al., 2013; Ma et al., 2014; Bondage et al., 2016). T6SS is a molecular syringe that injects effectors such as DNAse and amidase into target cells to kill them (Ma et al., 2014; Bondage et al., 2016). In the *Agrobacterium* C58 strain, this system is induced in acidic conditions by the ChvG/I two-component system and repressed by ExoR (Wu et al., 2012; Heckel et al., 2014). The T6SS expression is also induced upon high level of intracellular cyclic di-GMP (McCarthy et al., 2019). Interestingly, a *P. aeruginosa* T6SS was shown to inhibit “*A. fabrum*” growth *in vitro* (Ma et al., 2014). However, after co-infiltration in leaves, wild-type “*A. fabrum*” cells of strain C58 outcompeted *P. aeruginosa* whereas T6SS mutants were unable to do so (Ma et al., 2014). Thus, “*A. fabrum*” T6SS is efficient *in planta* but not *in vitro*. This suggests that plant compounds induce the expression of “*A. fabrum*” T6SS genes and/or affect *P. aeruginosa* competitive properties.

**Figure 2:**
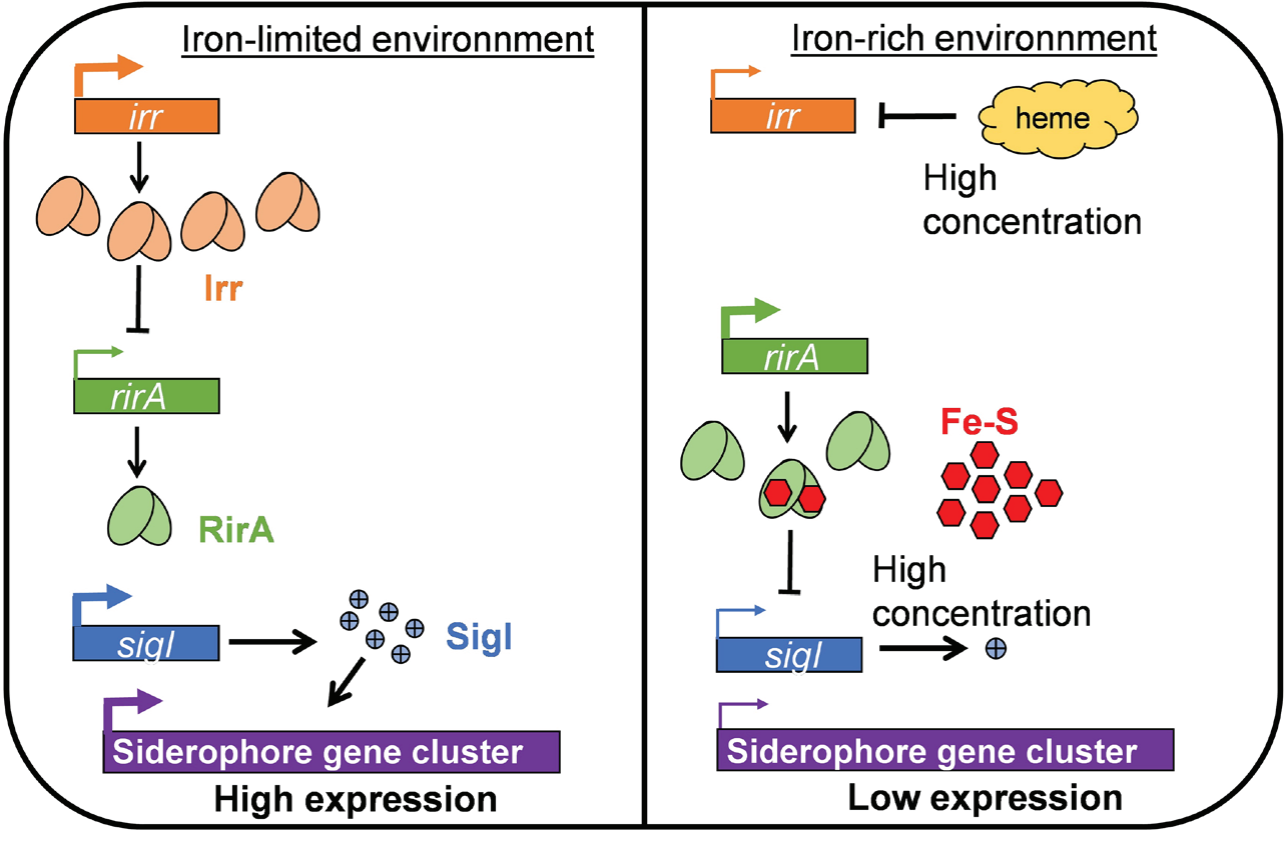
Model of the regulation of siderophore gene cluster expression according to iron availability. In an iron-limited environment, due to the low heme and Fe-S concentrations, the Irr protein is abundant and represses *rirA*. Consequently, SigI, which is under the negative control of RirA, is expressed and can induce siderophore synthesis. In an iron-rich environment, Irr is negatively regulated by the high heme concentration. In these conditions, RirA is abundant and its binding to Fe-S allows for the repression of *sigI,* which leads to the very low expression of siderophore biosynthesis genes.

The ability to catch and/or sequester iron is important for competition under low-iron conditions, and iron scavenging is known as a plant defense mechanism against phytopathogens (Aznar et al., 2014; Niehus et al., 2017). In this context, siderophores, the most widespread bacterial system for iron acquisition, are certainly determining for the competitive colonization of tumors (Aznar et al., 2014; Verbon et al., 2017). The different *Agrobacterium* species seem able to produce distinct siderophores, but an operon involved in siderophore biosynthesis has so far only been characterized in *Agrobacterium* strain C58 (Rondon et al., 2004). Deletion of this large gene cluster (nearly 50 kb) abolished the bacterial capacity to survive in an iron-limited medium (Rondon et al., 2004; Liu et al., 2016). However, a tight control of siderophore gene expression is needed because this expression can be metabolically costly (Miethke and Marahiel, 2007; Harrison et al., 2008; Verbon et al., 2017). Regulatory proteins controlling siderophore biosynthesis are RirA, Irr, and SigI, a sigma factor influenced by heme and Fe-S concentrations (Figure 2; Qi et al., 1999; Ngok-Ngam et al., 2009; Hibbing and Fuqua, 2011). The exact mechanism of regulation by SigI and the structure of “*A. fabrum*” C58 siderophore remain unknown. The global regulator LsrB was also recently shown to be involved in iron homeostasis by positively regulating siderophore biosynthesis genes (Tang et al., 2018). Among the microorganisms highly adapted to the plant environment, some possess multiple receptors and ABC transporters; some of these make the uptake of heterologous siderophores possible (Loper and Buyer, 1991; Lemanceau et al., 2009; Berendsen et al., 2015). The *Agrobacterium* strain C58 genome contains three ABC transporters annotated as useful for iron uptake (*atu0408–atu0406; atu2473–atu2476*; *atu5311–atu5316*; respectively on circular chromosome, linear chromid, and At plasmid); they appear to be up-regulated in tumors as compared to culture media (González-Mula et al., 2018). Information about the compound actually transported in *Agrobacterium* or about the specificity of those transporters is unavailable, but it is tempting to speculate that these compounds are important for the uptake of heterologous siderophores and for bacterial fitness within tumors.

## Concluding Remarks

In tumors, agrobacteria are exposed to plant defense reactions, hypoxic and drought conditions, as well as competition with other tumor dwellers. Even if the opine concept explains a major part of the *Agrobacterium*-plant interaction, it cannot solely justify *Agrobacterium* maintenance and competitiveness in tumors. The high tumor-colonizing capacity of *Agrobacterium* is likely to be conferred by its ability to survive stresses encountered in tumors, to kill competitors, its metabolic capacities, and efficient resource uptake.

Unfortunately, the tumor metabolites content is sparsely described. To date, only a few untargeted metabolites studies provide valuable but limited information since they were performed at different stages (i.e., 4- to 5-week-old tumors) on different plant species (*B. rapa*, *A. thaliana*, *S. lycopersicum*) and with different analytical methods (Deeken et al., 2006; Simoh et al., 2009; González-Mula et al., 2019). The integration of metabolite and plant gene expression analyses contributes to a better understanding of the changes that occur during tumor development and of the way the plant faces such modifications (Deeken et al., 2006). Moreover, integrated analyses of the two partners would be essential to understand the different aspects of the long-term interaction inside the tumor.

Even if *Agrobacterium* has been the subject of many functional studies, genetic characterizations have mostly been focused on virulence genes, the factors influencing T-DNA integration, and the efficiency of plant genetic transformation. There is growing interest in the characterization of the genetic determinants that allow for rhizosphere colonization, and more recently for the transition between the rhizosphere and pathogenic lifestyles. Many bacterial functions highlighted in this review are indeed found in non-tumor environments. EPS and siderophores are usually described as essential for root colonization (as reviewed by Dessaux and Faure, 2018; Matthysse, 2018). Moreover, the import and catabolism of melibiose, raffinose, stachyose, and galactinol have recently been showed to confer a competitive advantage for rhizosphere colonization (Meyer et al., 2018b). The ability of *Agrobacterium* to synthesize hormones might modify the root architecture, as observed for plant-growth-promoting rhizobacteria (Vacheron et al., 2013), thus providing a favorable habitat to the bacterium. Therefore, besides the characterization of new genetic clusters, an additional effort is needed not only to decipher the role of these clusters in bacterial virulence (their tumor-inducing ability) as often tested, but also to evaluate their role in colonization, persistence, and in the rhizosphere-to-tumor transition. This would provide further insights into key determinants of *Agrobacterium* ecology, as well as valuable information on disease persistence and dissemination.

## Author Contributions

CL, LV, TM, and CT-M conceived the idea and designed the outlines of the article. TM, CT-M, LV, FW-D, and CL wrote the manuscript. GC and IK contributed in revising manuscript. All authors listed have made a substantial, direct, and intellectual contribution to the work, and approved it for publication.

### Conflict of Interest Statement

The authors declare that the research was conducted in the absence of any commercial or financial relationships that could be construed as a potential conflict of interest.
